# Protein Phosphatase 2A: More Than a Passenger in the Regulation of Epithelial Cell–Cell Junctions

**DOI:** 10.3389/fcell.2019.00030

**Published:** 2019-03-06

**Authors:** Diana Schuhmacher, Jean-Marie Sontag, Estelle Sontag

**Affiliations:** ^1^School of Biomedical Sciences and Pharmacy, Faculty of Health and Medicine, University of Newcastle, Callaghan, NSW, Australia; ^2^Hunter Medical Research Institute, New Lambton Heights, NSW, Australia

**Keywords:** adherens junction, dephosphorylation, desmosome, polarity, PP2A, signaling, tight junction

## Abstract

Cell–cell adhesion plays a key role in the maintenance of the epithelial barrier and apicobasal cell polarity, which is crucial for homeostasis. Disruption of cell–cell adhesion is a hallmark of numerous pathological conditions, including invasive carcinomas. Adhesion between apposing cells is primarily regulated by three types of junctional structures: desmosomes, adherens junctions, and tight junctions. Cell junctional structures are highly regulated multiprotein complexes that also serve as signaling platforms to control epithelial cell function. The biogenesis, integrity, and stability of cell junctions is controlled by complex regulatory interactions with cytoskeletal and polarity proteins, as well as modulation of key component proteins by phosphorylation/dephosphorylation processes. Not surprisingly, many essential signaling molecules, including protein Ser/Thr phosphatase 2A (PP2A) are associated with intercellular junctions. Here, we examine how major PP2A enzymes regulate epithelial cell–cell junctions, either directly by associating with and dephosphorylating component proteins, or indirectly by affecting signaling pathways that control junctional integrity and cytoskeletal dynamics. PP2A deregulation has severe consequences on the stability and functionality of these structures, and disruption of cell–cell adhesion and cell polarity likely contribute to the link between PP2A dysfunction and human carcinomas.

## Introduction

Cell–cell adhesion is critical for the biogenesis and maintenance of epithelial tissue. Its deregulation is a prominent feature of many disorders, including carcinomas (Salvador et al., 2016), asthma (Wittekindt, 2017), and inflammatory bowel diseases (Lee, 2015). It is maintained by three major junctional complexes: desmosomes, adherens junctions (AJs), and tight junctions (TJs). Each comprises a plethora of proteins that drive junctional assembly and dynamics; their expression and activity must therefore be precisely regulated in order to maintain homeostasis. In this context, phosphorylation and dephosphorylation processes that derive from the concerted action of protein kinases and phosphatases, play a critical role in governing junctional protein–protein interactions and function. Here, we focus on reviewing the important contribution of protein Ser/Thr phosphatase 2A (PP2A), a major signaling enzyme, in the homeostatic regulation of epithelial intercellular junctions. We also discuss how deregulation of PP2A-dependent cell–cell adhesion and polarity could contribute to disease, with a particular emphasis on cancer.

## PP2A: a Family of Essential Signaling Molecules

**FIGURE 1:**
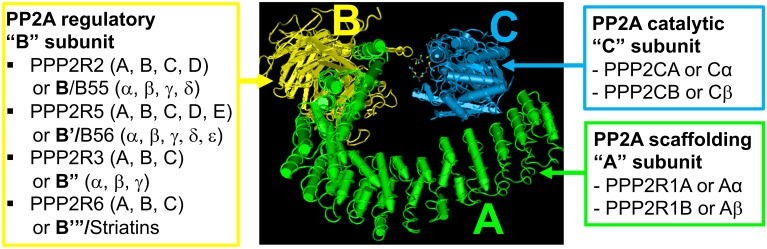
Schematic structure of a classical PP2A holoenzyme. Catalytic (C or PPP2C), structural (A or PPP2R1), and regulatory (B or PPP2R) subunits make up the typical mammalian heterotrimeric PP2A holoenzyme. There are several families of B subunits, and multiple subunit isoforms (denoted A, B,…or α, β,…), resulting in a diversity of PP2A holoenzymes with distinct enzymatic activity and substrate specificity. Other atypical subunits and regulatory proteins not shown here can also associate with PP2A C (Sents et al., 2013). The PP2A/B structure (Xu et al., 2008) was adapted from https://www.ncbi.nlm.nih.gov/Structure/pdb/3DW8.

PP2A is a family of major, evolutionarily conserved enzymes that collectively regulate most signal transduction pathways and cellular processes (Sontag, 2001; Seshacharyulu et al., 2013). The typical mammalian PP2A holoenzyme is a heterotrimer made up of a catalytic (C or PPP2C), structural (A or PPP2R1), and regulatory (B-type) subunit (Figure 1). There are two isoforms (α and β) of the A and C subunits, and four known families of regulatory “B-type” subunit (B or PPP2R2; B′ or PPP2R5; B^′′^ or PPP2R3; B^′′′^ or PPP2R6), each containing several isoforms. The modulation of PP2A biogenesis, activity, and targeting are highly complex and still poorly understood processes that ensure PP2A substrate specificity. They are controlled in part by binding of a specific B subunit and other regulators to the PP2A (AC) core enzyme, as well as by post-translational modifications (Methylation, phosphorylation, ubiquitination) of the catalytic subunit (Sents et al., 2013). The prevalent position of PP2A in cell signaling and homeostasis is illustrated by the preferential targeting of PP2A by numerous pathogenic viruses and parasites (Garcia et al., 2000). Of particular interest herein is Simian Virus 40 small tumor antigen (SV40 st); this early viral protein binds to the PP2A A subunit via the same binding site used by the regulatory B subunit, resulting in changes to PP2A substrate specificity and cell transformation (Sontag et al., 1993; Moreno et al., 2004; Cheng et al., 2009). PP2A activity is also targeted by a variety of naturally occurring toxins, including okadaic acid (OA), microcystin-LR, calyculin-A, cantharidin, and fostriecin (Sontag, 2001). These molecules inhibit all PP2A isoforms by binding to the catalytic subunit. Importantly, they also inhibit other Ser/Thr phosphatases (PP1, PP4, PP5, and PP6) at concentrations typically used in cells and *in vivo*, which can confuse the interpretation of their effects (Swingle et al., 2007).

Based on the central role of the PP2A family in the regulation of crucial cellular processes, it comes to no surprise that PP2A dysfunction is associated with human diseases, including neurodegenerative disorders (Sontag and Sontag, 2014), heart disease, diabetes (Baskaran and Velmurugan, 2018), asthma (Kobayashi et al., 2011), and cancer (Seshacharyulu et al., 2013; Thompson and Williams, 2018). Altered PP2A subunit expression occurs in many human malignancies. For instance, PP2A Cα (Singh et al., 2008) and Bα (Cheng et al., 2011) subunits are downregulated in prostate cancer, while the A subunit is downregulated in breast and lung carcinomas (Calin et al., 2000). Furthermore, the endogenous PP2A inhibitors, CIP2A and SET, are upregulated in many cancers (Fujiki et al., 2018). As such, PP2A has been proposed to be a tumor suppressor (Seshacharyulu et al., 2013). However, PP2A subunits are also upregulated in certain cancers. For instance, elevated levels of Bα that sustain oncogenic signaling and promote metastasis are found in pancreatic cancer (Hein et al., 2016). Moreover, PP2A inactivation induces apoptosis in several cancers (Kiely and Kiely, 2015). Thus, changes in cellular PP2A subunit composition could promote or inhibit tumorigenesis depending on cell context. In any case, there is increasing evidence that deregulation of PP2A-dependent cell–cell adhesion could contribute or underlie many of these pathological processes, as more specifically discussed thereafter.

## Desmosomes are Emerging Targets of PP2A

### PP2A Inhibition Is Associated With Desmosomal Disruption

**FIGURE 2:**
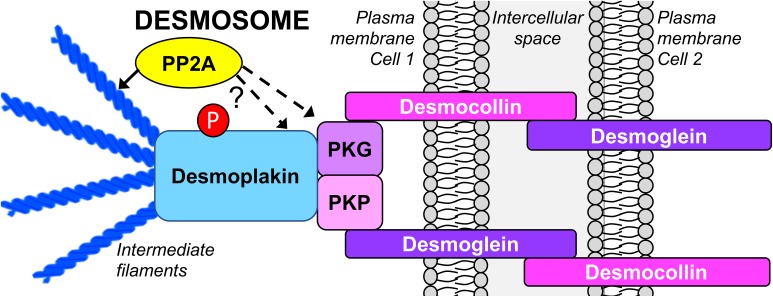
Schematic organization of the epithelial desmosome targeted by PP2A. There is emerging evidence that inhibition of PP2A promotes desmosomal disassembly by directly affecting the phosphorylation state of desmoplakin and plakoglobin, and indirectly, by inducing the reorganization of the IF cytoskeleton. PKG, plakoglobin; PKP, plakophilin.

Desmosomes represent major intercellular adhesive junctions at basolateral membranes of epithelial cells. They “bolt” cells together by attaching to the intermediate filament (IF) cytoskeleton of apposing cells, forming a structure that allows cells to resist breakage upon mechanical stress, such as in the heart and the skin. Desmosomes consist of proteins from three families: desmosomal cadherins, armadillo, and plakin. The transmembrane desmosomal cadherins, desmocollin and desmoglein, form a complex with plakophilin and plakoglobin, as well as desmoplakin, which links the desmosome to the IF cytoskeleton (Figure 2) (Zhou et al., 2017). While desmosome formation is calcium-dependent, several studies suggest that Ser/Thr phosphorylation of key desmosomal proteins negatively modulates desmosomal assembly and stability. For instance, protein kinase C (PKC) and protein kinase A-mediated Ser-phosphorylation of desmoplakin results in its dissociation from the IF cytoskeleton, and decreased cell–cell contacts (Stappenbeck et al., 1994; Amar et al., 1999). Furthermore, plakoglobin is in a dephosphorylated state in the desmosomal complex (Yin and Green, 2004; Pasdar et al., 2005).

Studies relying on inhibitory toxins have implicated PP2A-like phosphatase activity in the regulation of desmosomal assembly and stability. Incubation of hepatocytes with microcystin-LR correlates with hyperphosphorylation and redistribution of desmoplakin away from areas of cell–cell contact into the cytosol and nucleus, concomitant with desmosomal disruption (Toivola et al., 1997). Likewise, OA inhibits desmosomal assembly in Madin-Darby Canine Kidney (MDCK) cells (Pasdar et al., 2005). Expression of SV40 st in human embryonic kidney cells reduces plakoglobin expression levels in a PP2A-dependent manner (Moreno et al., 2004). While not demonstrated, we hypothesize that this effect is mediated by enhanced phosphorylation of plakoglobin as a result of PP2A inhibition; this may lead to detachment of plakoglobin from desmosomes, subsequent lysosomal degradation of soluble plakoglobin, and ultimately desmosomal disassembly.

### What Could Be the Functional Significance of PP2A-Dependent Desmosomal Regulation?

Genetic mutations and autoimmune events targeting desmosomal proteins are causal to diseases manifested by severe skin and heart defects (Hatzfeld et al., 2017). Downregulation of various desmosomal proteins also occur in acquired disorders associated with epithelial barrier dysfunction. For instance, there is a loss of desmoplakin in human lung epithelial cells exposed to pollutants (Thevenot et al., 2013), and reduced levels of desmoglein 2 in inflammatory bowel diseases (Spindler et al., 2015). Profound changes in the expression levels of desmosomal proteins are highly relevant to the progression of many carcinomas. The downregulation of desmocollin 3 in breast, desmocollins 1 and 3 in lung (Zhou et al., 2017), desmoglein 1 in head and neck squamous cell (Wong et al., 2008), desmoglein 2 in prostate (Barber et al., 2015), or desmocollin 2 in pancreatic (Hamidov et al., 2011) cancer correlate with poor prognosis and shorter patient survival. Plakoglobin has been proposed to be a tumor suppressor based on the observation that SV40-transformed cells have decreased levels of plakoglobin that correlate with tumorigenicity (Simcha et al., 1996). Thus, PP2A-mediated deregulation of plakoglobin (Moreno et al., 2004) could contribute to SV40 st-dependent cell transformation. In contrast, increased expression of certain desmosomal proteins has been linked to carcinoma development. For instance, enhanced expression of desmoglein 3 promotes migration and invasion of epithelial A431 cancer cell lines (Zhou et al., 2017). Desmoglein 2 expression levels are increased in human colon adenocarcinomas and correlate with enhanced cell proliferation (Kamekura et al., 2014). These discrepancies illustrate that the deregulation of desmosomal proteins is a complex and tissue-specific process, and cancer cell-type PP2A dysfunction could lead to distinct functional consequences for desmosomal proteins and disease progression. While disruption of desmosomes is an important event in disease onset and development, much remains to be learned on the contribution of PP2A to the biogenesis and upkeep of this junctional structure.

## The Presence of PP2A at the Adherens Junction is Critical for Junctional Biogenesis and Stability

### PP2A Is Critically Required for Epithelial E-Cadherin-Mediated Cell–Cell Adhesion

**FIGURE 3:**
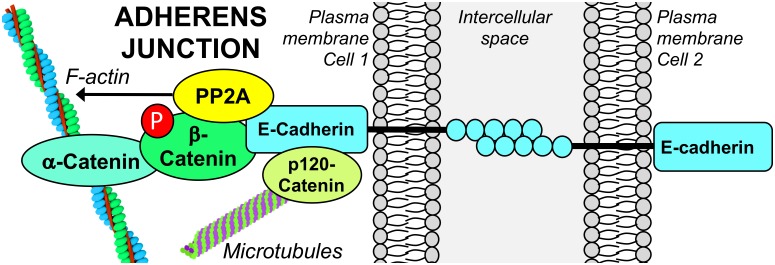
PP2A associates with the E-cadherin/β-catenin complex at the epithelial AJ. The functional integrity of PP2A is required for the stabilization of the E-cadherin/β-catenin complex at areas of cell–cell contact. PP2A activity is also involved in dephosphorylation of β-catenin and modulation of the actin cytoskeleton that is essential for AJ formation.

Adherens junctions are multiprotein complexes located directly under TJs, made up of transmembrane cadherins complexed to catenins (Figure 3). Classical cadherins are vital for the dynamics of cell–cell contact formation and remodeling. The formation of AJs is calcium-dependent; binding of extracellular calcium stimulates cadherin–cadherin interactions by reinforcing the cadherin extracellular domains. In turn, this enables junctional homophilic interactions, and recruitment of β-catenin and p120-catenin to the cadherin complex. AJ linkage to the actin cytoskeleton is also essential for cell adhesion. The actin-binding protein, α-catenin, acts as an essential physical linker between the cadherin/β-catenin complex and the actin cytoskeleton (Coopman and Djiane, 2016). AJ assembly and stability is dependent on Ser/Thr phosphorylation of key components. Phosphorylation of E-cadherin (Epithelial cadherin/cadherin 1/CDH1) on Ser840, Ser846, and Ser847 within its β-catenin-binding region occurs during its biosynthesis and is required for proper intercellular junctional assembly and stability. Mutations of these Ser to non-phosphorylatable Ala residues prevent the interaction of E-cadherin with β-catenin, leading to endocytosis and lysosomal degradation of E-cadherin, and impaired cell–cell adhesion (McEwen et al., 2014). Phosphorylation of E-cadherin within its cytoplasmic domain by casein kinase 2 or glycogen synthase kinase-3β (GSK-3β) promotes its interaction with β-catenin, augmenting the strength of cell–cell adhesion (Lickert et al., 2000). In contrast, phosphorylation of E-cadherin at Ser846 by casein kinase 1 reduces β-catenin binding and enhances E-cadherin internalization (Dupre-Crochet et al., 2007). PKCδ-mediated phosphorylation of E-cadherin at Thr790 inhibits homophilic interactions between E-cadherin ectodomains and reduces its binding to β-catenin, leading to disruption of cell–cell adhesion (Chen et al., 2016). PKC-mediated phosphorylation of p120-catenin is also implicated in AJ disassembly (Sandoval et al., 2001; Xia et al., 2003). Together, these studies reinforce the concept that complex, site-specific Ser/Thr phosphorylation events critically modulate AJ protein interactions, thereby controlling AJ strength and the integrity of cell–cell contacts.

The involvement of cellular Ser/Thr phosphatase activity in AJ regulation is supported by several studies relying on the utilization of inhibitory toxins. Incubation of keratinocytes with 0.5 μM OA induces the translocation of E-cadherin from the membrane to the cytosol, and a complete loss of cell–cell adhesion (Serres et al., 2000). Disrupted cell–cell adhesion and cell rounding also occur in OA-treated human mammary epithelial cells (Takahashi et al., 2006). However, since these studies utilized OA at high concentrations that also inhibit PP1 (Swingle et al., 2007), it is not clear whether these effects were mediated by PP2A. Indeed, in OA- or calyculin A-treated keratinocytes and fibroblasts, the loss of cell–cell contacts appeared to be preferentially mediated by PP1 (Serres et al., 1997). However, incubation of breast cancer cells with 50 nM OA -a lower concentration that typically does not inhibit PP1- ultimately results in a significant decrease in the cellular content of E-cadherin and loss of cell–cell adhesion (Malaguti and Rossini Gian, 2002). While these later studies point to a role of PP2A in the regulation of AJ proteins, it is worth mentioning that low concentrations of OA can inhibit other Ser/Thr phosphatases besides PP2A (Swingle et al., 2007). The specific contribution of PP2A in AJ regulation was demonstrated using more targeted approaches. PP2A Cα subunit, E-cadherin and β-catenin are found in a complex at the plasma membrane during early embryonic mouse development. Knockdown of Cα induces a loss of E-cadherin and β-catenin in mouse embryos (Gotz et al., 2000), and the dissolution of the E-cadherin/β-catenin complex in various epithelial cell lines (Su et al., 2008), indicating that PP2A is critical for stabilizing the membrane-bound E-cadherin/β-catenin adhesion complex. PP2A A and C subunits also co-localize with E-cadherin/β-catenin complexes at cell–cell adhesion sites in confluent, non-malignant human mammary epithelial cells. Silencing of the A subunit and OA treatment result in a loss of cell–cell adhesion. Yet, the underlying mechanism does not involve a direct effect on formation of the E-cadherin/catenin complexes, but rather the indirect endocytosis of E-cadherin from the cell surface following dissociation of IQGAP1 from the complex (Takahashi et al., 2006). Likewise, reduced levels of PP2A A subunit are associated with internalization of E-cadherin in human breast cancer cell lines (Suzuki and Takahashi, 2006). Interestingly, hypoglycosylated E-cadherin has a high affinity for PP2A, and is preferentially recruited to β-catenin complexes in human epidermoid carcinoma cells, thereby stabilizing intercellular adhesion (Jamal et al., 2009). Together, these studies clearly indicate that the functional integrity of PP2A is essential for proper formation of E-cadherin/β-catenin complexes integral to the AJ. However, there are still unresolved questions on the molecular mechanisms mediating the internalization of E-cadherin. Furthermore, the specific PP2A enzyme associated with the epithelial AJ has yet to be identified.

### The Downregulation of E-Cadherin/β-Catenin Adhesion Complexes Is Associated With Cancer Metastasis

As stated above, PP2A dysfunction in disease could ultimately lead to a loss of the E-cadherin/catenin adhesion complex. Notably, disruption of the E-cadherin/β-catenin complex is a common theme in pathological conditions, including inflammatory bowel disease (Zbar et al., 2004) and asthma (Nawijn et al., 2011). Of particular significance, the downregulation of E-cadherin is a prominent feature of many carcinomas, including breast (Vergara et al., 2015), squamous cell lung (Zhang et al., 2013), endometrial (Feng et al., 2013), and prostate (Barber et al., 2015) cancer. Indeed, the loss of E-cadherin is a hallmark of epithelial–mesenchymal transition (EMT), a process that promotes cancer metastasis by enhancing cell migration, invasion, and resistance to apoptosis (Lamouille et al., 2014). Accordingly, reduced E-cadherin expression levels typically correlate with indicators of more severe malignancy and poor prognosis (Feng et al., 2013; Zhang et al., 2013). In this context, it is worth mentioning that while the loss of E-cadherin is intimately linked to EMT, other cell-type specific cadherins become inappropriately expressed in cells that have acquired the mesenchymal phenotype. For instance, the downregulation of epithelial E-cadherin is counter balanced by the upregulation of mesenchymal N-cadherin (Neural cadherin/cadherin 2/CDH2). This aberrant switch in cadherin types during EMT has been shown to initiate pro-migratory signaling pathways and dynamically enhance the strength of cell–cell cohesion. These profound changes favor the formation of cancer cell collectives that subvert the tumor microenvironment, thereby promoting tumor cell migration and invasion (Shih and Yamada, 2012; Lamouille et al., 2014). As mentioned earlier, the deregulation of PP2A subunit composition and/or inhibition of its catalytic activity occur in several human carcinomas, and experimentally coincide with the internalization and downregulation of E-cadherin. Notably, during EMT induced by transforming growth factor-β in A549 lung adenocarcinoma cells, decreased expression of PP2A not only correlates with reduced E-cadherin levels, but also with enhanced expression of N-cadherin (Kim et al., 2015). As observed with N-cadherin, expression of mesenchymal OB-cadherin (Osteoblast cadherin/cadherin-11/CDH11) is associated with EMT and tumor progression, and is typically found in poorly differentiated and invasive carcinomas with poor prognosis (Assefnia et al., 2014). Interestingly, *CDH11* gene expression is strongly suppressed by expression of polyomavirus small tumor antigen (Klucky et al., 2004). Like SV40 st, this viral protein primarily targets and deregulates PP2A to force quiescent host cells to enter into the S-phase of the cell cycle, thereby allowing viral replication (Garcia et al., 2000). These findings raise the possibility that deregulation of PP2A can also influence cellular levels of OB-cadherin. Lastly, increased expression of P-cadherin (Placental cadherin/cadherin-3/CDH3) has also been described in certain advanced carcinomas, wherein E-cadherin is again characteristically downregulated. It is noteworthy that overexpression of PME-1, an enzyme that demethylates and inactivates PP2A, correlates with the loss of E-cadherin and presence of P-cadherin in aggressive endometrial cancer (Wandzioch et al., 2014). Altogether, these findings suggest the existence of a compelling relationship between deregulation of PP2A, altered expression levels of different cadherins, EMT and metastasis, which merits further investigation. More studies are also necessary to determine whether specific PP2A holoenzymes can directly interact with and regulate cadherins.

Mechanistically, the loss of E-cadherin not only leads to the dissociation of the membrane-bound E-cadherin/β-catenin complex and disruption of AJs, but also to the activation of major cancer-promoting signaling pathways that upregulate transcription factors linked to EMT (Coopman and Djiane, 2016). Notably, it is associated with alterations in β-catenin subcellular localization and increased β-catenin dependent transcription. Indeed, this is due to the dual regulatory role of distinct cellular β-catenin pools: β-catenin not only functions in cell adhesion as part of the stabilized, membrane-associated cadherin/catenin AJ complex, but also in the nucleus as a regulator of gene transcription in the Wnt signaling pathway. The localization of β-catenin is dependent on its phosphorylation state. In absence of Wnt signal, cytoplasmic β-catenin is constantly phosphorylated and targeted for ubiquitin-mediated proteasomal degradation, as a result of the action of a functional β-catenin destruction complex. When dephosphorylated, β-catenin translocates from membrane and/or cytoplasmic pools to the nucleus, wherein it controls expression of genes affecting growth, proliferation and apoptosis. Deregulation of the Wnt/β-catenin pathway results in an overabundance of nuclear β-catenin, and aberrant activation of Wnt/β-catenin target genes that promote malignant cell transformation (Jamieson et al., 2012; Thompson and Williams, 2018). Thus, deregulation of β-catenin and/or AJs may be problematic in more ways than one. Not only does accumulation of β-catenin in the nucleus stimulate carcinogenesis, it also results in the removal of β-catenin from the AJ resulting in a loss of cell–cell adhesion, thus promoting EMT and metastasis.

PP2A isoforms play an important but complex role in the regulation of Wnt signaling (Thompson and Williams, 2018). The PP2A core enzyme is responsible for the rapid dephosphorylation of free, cytoplasmic phospho-β-catenin (Su et al., 2008). In human pancreatic cancer cells, PP2A inhibition increases β-catenin phosphorylation and promotes its degradation (Wu et al., 2014). Likewise, knockdown of PP2A Cα subunit in cells (Su et al., 2008) and *in vivo* (Gotz et al., 2000) results in hyperphosphorylation of β-catenin, and excessive degradation of both cytoplasmic and membrane-bound β-catenin. In HEK293T and SV480 epithelial cells, the PP2A Bα (or PPP2R2A) subunit directly binds to the cytoplasmic β-catenin associated with the axin complex that functions in Wnt signaling. Overexpression of the PP2A Bα subunit enhances Wnt signaling, while its knockdown results in β-catenin phosphorylation and decreased Wnt signaling (Zhang et al., 2009). In contrast, other studies have implicated PP2A/B′ holoenzymes as the prevalent regulator of Wnt/β-catenin signaling. The B′ (B56) subunit directly associates with adenomatous polyposis coli (APC) belonging to the Wnt-regulated, axin/GSK-3β signaling complex (Seeling et al., 1999). In contrast to Bα (Zhang et al., 2009), overexpression of B′ reduces the abundance of β-catenin and inhibits Wnt signaling (Seeling et al., 1999). Thus, the ability of PP2A to directly dephosphorylate β-catenin, thereby regulating its degradation in the Wnt signaling pathway, takes central stage in indirectly regulating the cellular levels of β-catenin available for recruitment and stabilization at the AJ. It is noteworthy that, by differentially affecting β-catenin regulation, cancer-related changes in the expression of specific PP2A subunits could have indirect and opposite consequences on AJ biogenesis. In any case, underlying pathways need to be fully elucidated.

## PP2A Plays a Critical Role in TJ Homeostasis and Cell Polarity

### Cellular Pools of PP2A Are Targeted to the TJ

**FIGURE 4:**
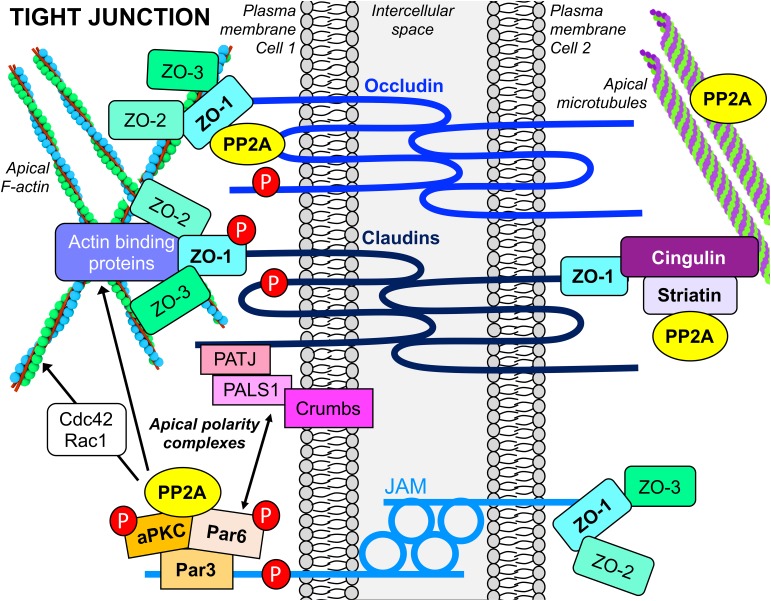
PP2A interacts with apical TJ and polarity complexes in epithelial cells. PP2A enzymes are associated with the multifunctional apical TJ structure, a complex assembly of transmembrane, scaffolding, cytoskeletal and signaling proteins. PP2A also interacts with proteins of apical polarity complexes, which are essential for the establishment of epithelial cell polarity. PP2A activity is implicated in dephosphorylation of several TJ proteins (red P circles) that regulate TJ assembly and maintenance. PP2A enzymes also influence the formation and stability of TJs by modulating actin dynamics via their action on actin-regulatory proteins, as well as binding to and regulating microtubule stability.

The TJ is a multifunctional, multiprotein complex located at the most apical point of cell–cell contacts (Figure 4). Besides its role in intercellular adhesion, it serves as a signaling platform, a “gate” that controls the paracellular movement of ions and molecules, and a “fence” that determines the asymmetric distribution of proteins and lipids between the apical and basolateral cell compartments, which is fundamental for cell polarity. The TJ consists of integral transmembrane proteins [occludin, claudins, and junctional adhesion molecules (JAMs)] that bind to scaffolding proteins belonging to the zonula occludens (ZO) family. The ZO adaptor proteins act as a bridge to join the cytoplasmic domains of the transmembrane proteins with the actin cytoskeleton, and other actin-binding and TJ proteins (Zihni et al., 2016). Another important scaffolding TJ protein is cingulin, which associates with microtubules. It plays a critical role in organizing and tethering the apical microtubule network to the TJ (Vasileva and Citi, 2018). Significantly, apical PAR and CRUMBS polarity complexes are responsible for TJ construction and maintenance, and the establishment of epithelial cell apicobasal polarity (Wen and Zhang, 2018).

In epithelial cells and human tissue, pools of PP2A/Bα holoenzymes are specifically associated with mature TJs (Nunbhakdi-Craig et al., 2002). Expression of SV40 St in MDCK cells, which impedes binding of Bα to the PP2A core enzyme, prevents the proper targeting of PP2A to the TJ (Nunbhakdi-Craig et al., 2003). The expression of Bα is also critical for barrier acquisition during late embryonic epidermal development; activation of Akt signaling and downstream PP2A/Bα-mediated dephosphorylation of c-Jun are essential for this process (O’Shaughnessy et al., 2009). PP2A/Bα co-immunoprecipitates with occludin, claudin-1, and ZO-1 (Nunbhakdi-Craig et al., 2002) and directly binds to occludin *in vitro* (Seth et al., 2007) (Figure 4). Recent work has also demonstrated that striatin, which can serve as a regulatory subunit of PP2A (Figure 1), co-localizes with TJ proteins (ZO-1, occludin, and cingulin), but not E-cadherin, in several polarized epithelial cell lines (Lahav-Ariel et al., 2018). *In vitro* assays showed that striatin forms a complex with the PP2A C subunit and the TJ protein, cingulin, but not E-cadherin, suggesting that striatin could target PP2A to multi-protein TJ-associated cingulin complexes (Figure 4). Striatin also co-localizes with APC at the TJ, and directly binds to APC *in vitro*. Notably, occludin co-immunoprecipitates with both APC and striatin. While the PP2A Cα subunit directly associates with occludin *in vitro* (Seth et al., 2007), further work is needed to investigate whether striatin serves to anchor the PP2A core enzyme to the APC/striatin/occludin complex at the TJ.

### Direct PP2A-Mediated Dephosphorylation of TJ Proteins Negatively Regulates TJ Biogenesis, Stability, and Function

As observed with other junctions, TJ dynamics are modulated by reversible phosphorylation of its individual proteins. For example, occludin becomes highly phosphorylated on Ser/Thr residues during TJ assembly and maturation, while TJ disassembly promotes its dephosphorylation (Rao, 2009; Manda et al., 2018). In that regard, the targeting of PP2A/Bα enzymes to the TJ complex means that they are ideally positioned to modulate the dephosphorylation of TJ protein substrates. Indeed, PP2A plays a major role in TJ biogenesis and stability by dephosphorylating key TJ proteins. PP2A directly dephosphorylates occludin, ZO-1 and claudin-1 (Nunbhakdi-Craig et al., 2002; Seth et al., 2007; Dunagan et al., 2012). PP2A activity negatively regulates calcium-dependent TJ assembly in MDCK and Caco-2 cells, at least in part by promoting the dephosphorylation of occludin (Nunbhakdi-Craig et al., 2002; Seth et al., 2007). Expression of the PP2A catalytic subunit in MDCK cells, which enhances overall PP2A activity, induces deficits in TJ assembly that coincide with the cytoplasmic accumulation of ZO-1, occludin, and claudin-1 in a dephosphorylated (Ser/Thr) state (Nunbhakdi-Craig et al., 2002). Conversely, PP2A inhibition accelerates calcium-mediated TJ assembly by promoting the phosphorylation of occludin, ZO-1 and claudin-1 and their subsequent recruitment to areas of cell–cell contacts. The ability of OA to hasten junctional organization is further supported by the concomitant increase in transepithelial electrical resistance and paracellular barrier function measured in OA-treated cells (Nunbhakdi-Craig et al., 2002; Seth et al., 2007). Downregulation of the PP2A catalytic subunit similarly promotes TJ assembly in Caco-2 cells (Seth et al., 2007). The critical role played by PP2A/Bα holoenzymes during junction formation is further supported by the presence of TJ deficits in MDCK cells expressing SV40 st, which prevents targeting of PP2A/Bα to the TJ (Nunbhakdi-Craig et al., 2003).

Yet, the exact spatio-temporal mechanisms by which PP2A/Bα and other PP2A holoenzymes may be recruited to the desmosome, AJ, and TJ during cell junction biogenesis and maturation are completely unclear. It is likely that specific signal-mediated events control the formation of PP2A-containing protein scaffolds during junction formation, by inducing post-translational changes in PP2A subunits, and/or affecting the binding of a plethora of activators, inhibitors and/or adaptor proteins. In general, how the cellular (re)distribution of individual PP2A isoforms is precisely modulated by signaling events is not very well-understood due to the intricacy of these intertwined regulatory processes. Interestingly, it has been reported that during junctional biogenesis, hypoglycosylation of E-cadherin promotes the recruitment of PP2A to the AJ, resulting in decreased association of PP2A with ZO-1 and claudin-1; in turn, this may promote their phosphorylation and drive TJ assembly (Nita-Lazar et al., 2010). Likewise, protein interactions between PP2A and occludin are decreased during TJ formation (Seth et al., 2007). Recent studies also suggest that striatin species critically participate in early biogenesis of cell–cell junctions, via an uncharacterized mechanism involving E-cadherin; indeed, the loss of E-cadherin induces the cytoplasmic redistribution of striatin (Lahav-Ariel et al., 2018). However, like PP2A/Bα (Nunbhakdi-Craig et al., 2002), PP2A/striatin (Lahav-Ariel et al., 2018) does not bind to or co-localize with E-cadherin at the AJ. Whether PP2A is required for the function of striatin in the initial formation of cell–cell contacts, and how striatin may affect PP2A function in that regard remain to be investigated.

PP2A also plays an important role during TJ disassembly. Disassembly of cell–cell junctions following removal of extracellular calcium results in a significant decrease in the Thr phosphorylation of occludin in Caco-2 cells; this is mediated in part by increased association of occludin with PP2A, and its subsequent dephosphorylation (Seth et al., 2007). Further studies have shown that exposure of Caco-2 cells to acetaldehyde (Dunagan et al., 2012) or hydrogen peroxide (Sheth et al., 2009) can enhance the interaction of PP2A and occludin in a tyrosine kinase dependent manner, resulting in PP2A-mediated dephosphorylation of occludin, translocation of occludin and ZO-1 from intercellular junctions to the cytosol, and TJ disruption and leakiness. SV40 st-mediated loss of TJ-associated PP2A/Bα is also associated with altered distribution and downregulation of occludin, ZO-1 and claudin-1, and enhanced TJ disruption and permeability (Nunbhakdi-Craig et al., 2003). Incubation of Sertoli epithelial cells with the PP2A inhibitor, microcystin-LR, induces similar effects; reduced ZO-1, occludin, and claudin expression levels in these cells result from activation of the Akt/Snail signaling pathway that controls transcription of TJ proteins (Zhou et al., 2018). During epidermal development, Akt-dependent PP2A/Bα expression is critical for the cell surface localization of claudin-1 and its association with occludin (Youssef et al., 2013), and the membrane recruitment of ZO-1 (Gerner et al., 2013). Knockdown of Bα induces the cytoplasmic accumulation of claudin-1 and associated defects in paracellular epidermal barrier function (Youssef et al., 2013). Expression of SV40 st in human embryonic kidney cells also influences the expression of claudin-11 and JAM-1 (Moreno et al., 2004).

Thus, PP2A/Bα holoenzymes are key components and regulators of the functional integrity of TJs. Nevertheless, further studies are needed to fully understand the spatiotemporal mechanisms that control the compartmentalization of PP2A/Bα and other holoenzymes during junctional assembly and disassembly. It will also be important to determine whether other PP2A isoforms act in a concerted or independent manner with PP2A/Bα to regulate junctional dynamics.

### The PP2A/aPKC Signaling Module Plays an Important Role in TJ Formation and Maturation, and Development of Epithelial Cell Polarity

In MDCK cells, PP2A/Bα holoenzymes also bind to atypical PKC (aPKC) (Nunbhakdi-Craig et al., 2002). This association was confirmed in *Drosophila*, wherein aPKC was found in a complex with Twins, the PP2A B subunit homolog (Chabu and Doe, 2009). aPKC plays a fundamental role in TJ biogenesis, homeostasis and cell polarity. Membrane-associated aPKC activity increases during cell–cell junction assembly and is involved in the phosphorylation of key TJ proteins, including occludin and ZO-1 (Stuart and Nigam, 1995). Significantly, PP2A/Bα holoenzymes regulate the activity and distribution of aPKC during TJ formation. Membrane-associated aPKC activity is inhibited in MDCK cells overexpressing the PP2A catalytic subunit. Conversely, during TJ assembly performed under serum-free conditions, OA promotes the activating phosphorylation of aPKC at Thr410, resulting in accelerated accumulation of the kinase at junctional areas. Inhibition of aPKC blocks the ability of OA to enhance the redistribution of TJ proteins from the cytoplasm to areas of cell–cell contact during junctional biogenesis (Nunbhakdi-Craig et al., 2002). Likewise, membrane-associated occludin levels are decreased in a PP2A- and aPKC-dependent manner in hypoxic alveolar epithelial cells (Caraballo et al., 2011). Upon recruitment to areas of cell–cell contact, aPKC also directly interacts with and phosphorylates the transmembrane protein, JAM-A, at the Ser285 site; this promotes TJ formation, junctional maturation and establishment of the barrier in polarized epithelial monolayers. Notably, PP2A dephosphorylates JAM-A at Ser285, thereby counteracting aPKC (Iden et al., 2012). Thus, PP2A can negatively regulate TJ assembly and maturation by targeting both aPKC activity and JAM-A.

Epithelial cell polarization relies on the concerted activities of asymmetrically localized protein assemblies. Notably, aPKC belongs to and regulates the evolutionarily conserved PAR (Par-6/aPKC/Par-3) polarity complex (Figure 4). The initial recruitment of the PAR complex to the junctional region is required for TJ formation in vertebrates and the precise architecture and development of apicobasal polarity in epithelia. The assembly and defined spatial localization of the PAR complex is orchestrated by a highly complex web of protein–protein interactions between polarity and regulatory proteins, which are controlled by phosphorylation. In mammalian epithelial cells, Par-3 recruits Par6/aPKC to the apical membrane; upon cdc42 binding, Par-6 binds to and activates aPKC, which in turn phosphorylates Par-3, inducing its dissociation from the Par-6/aPKC complex. This allows the released Par6/aPKC complex to segregate into the apical domain, while the released Par-3 is repositioned slightly toward the lateral domain to define the apical/lateral boundary. Phosphorylation of Par-3 by Par-1 kinase has been shown to antagonize the association of Par-3 with aPKC, thereby affecting its targeting and function in cell polarity (Wen and Zhang, 2018).

There is accumulating evidence that PP2A is a conserved interactor and modulator of proteins in the PAR complex. PP2A/Bα isoforms interact with and negatively regulate the apical distribution of aPKC in epithelial cells (Nunbhakdi-Craig et al., 2002). In *Drosophila* dividing neuroblasts, both Twins (PP2A B subunit homolog) and Mts (PP2A C subunit homolog) are similarly required to exclude aPKC from the basal cortex, a process essential for controlling cell polarity and self-renewal during asymmetric division (Chabu and Doe, 2009). Moreover, Mts can antagonize aPKC signaling by associating with and dephosphorylating Par-6 (Ogawa et al., 2009). In this model, PP2A also directly binds to Bazooka (Par-3 homolog) via its A subunit, and dephosphorylates Par-3 at the Ser1085 targeted by the Par-1 kinase. The loss of PP2A function leads to complete reversal of neuroblast polarity. Indeed, the antagonistic function of PP2A and Par-1 kinase is a key determinant of Bazooka localization during *Drosophila* neuroblast division (Krahn et al., 2009). Whether PP2A associates with and regulates Par-6 or Par-3 proteins is yet to be confirmed in mammalian epithelial cells. Nevertheless, those studies point to an essential role of PP2A in directing cell polarity.

### PP2A-Dependent Deregulation of TJ Proteins Could Contribute to Cancer Development and Metastasis

As mentioned, PP2A dysfunction has dramatic consequences on the expression, distribution, regulation, and function of TJ and polarity proteins. Significantly, the disruption or complete demise of functional TJs, which results in compromised epithelial barrier integrity and loss of cell polarity, is a common feature of many pathologies. Diminished TJ integrity associated with reduced expression levels of integral TJ proteins, such as occludin, claudin-1, JAM-A and ZO-1, is observed in inflammatory bowel disease (Lee, 2015) and airway inflammation (Wittekindt, 2017). Downregulation of claudins, occludin, and ZO-1 are linked to tumor progression and metastasis in many carcinomas; in most instances, more pronounced decreased expression levels of these proteins directly correlate with cancer aggressiveness and poor patient survival (Runkle and Mu, 2013; Tabaries and Siegel, 2017). In support of a role of TJ proteins in tumorigenesis, many TJ proteins like ZO-1 act as tumor suppressors through their ability to function as signal transduction molecules at the plasma membrane and in the nucleus, where they control gene expression. Mice lacking specific TJ proteins develop a hyperproliferative phenotype. The loss of TJ proteins also promotes transformation and increased metastatic potential in experimental cell lines (Runkle and Mu, 2013; Tabaries and Siegel, 2017). Notably, the tumorigenic potential of some viral oncoproteins encoded by human adenovirus and papillomaviruses is closely related to their ability to disrupt TJs (Latorre et al., 2005). Accordingly, SV40 st-mediated TJ disruption likely contributes to its role in cell transformation (Sontag and Sontag, 2006).

However, it is intriguing that aberrant overexpression of many TJ (claudins and JAM-A) and polarity (Par-3, Par-6, and aPKC) proteins is also correlated with early tumorigenesis, carcinoma invasion, metastasis, and poor prognosis (Martin-Belmonte and Perez-Moreno, 2011; Zhao et al., 2014; Ruan et al., 2017; Tabaries and Siegel, 2017). Upregulation of these proteins is hypothesized to result in their mislocalization and dysfunction, leading to abnormal activation of tumor-promoting signaling pathways that sustain cell growth and survival, as well as dominant TJ defects that potentiate malignant transformation and metastasis. Thus, variable and intricate patterns of altered expression (either abnormal elevation or loss) of TJ and polarity proteins are present in carcinomas depending on cell type and stage. A deeper understanding of the role of these proteins in cancer initiation and progression is needed. Whether PP2A deregulation in cancer positively or negatively affects these processes has yet to be determined.

## Direct and Indirect Regulation of the Cellular Cytoskeleton by PP2A Also Contributes to Its Role in the Regulation of Cell–Cell Junctions

The proper organization of actin filaments (F-actin), microtubules, and IFs, is crucial for the formation and maintenance of cell–cell adhesion and cell polarity. By playing an important role in the regulation of cytoskeletal dynamics (Hoffman et al., 2017), PP2A has the potential to indirectly influence junctional assembly and stability.

### Deregulation of PP2A Promotes the Reorganization of the Epithelial Actin Cytoskeleton

The actin cytoskeleton provides a structural framework necessary for defining cell shape and polarity. The integrity of the actomyosin cytoskeleton is essential for cell junction biogenesis and stability; it is tightly maintained by a complex array of actin-binding, scaffolding and signaling molecules. Among key regulators are Rho, Rac, and Cdc42; these members of the Rho family of GTPases serve as molecular switches that control a wide variety of signal transduction pathways regulating actin cytoskeletal dynamics and associated cell functions (Arnold et al., 2017). Rho and Rac cooperate to establish cadherin-mediated cell–cell adhesion (Braga et al., 1997). Notably, PP2A mediates recruitment of the Ras GTPase-activating-like protein, IQGAP1, to Rac1-bound E-cadherin/catenin complexes, thereby promoting E-cadherin stabilization; deregulation of PP2A by OA, or silencing of the A subunit is associated with dissociation of IQGAP1, E-cadherin internalization, F-actin disassembly and AJ disruption (Takahashi et al., 2006). Blocking Rho function in polarized epithelial cells results in significant disruption of the apical F-actin cytoskeleton, with detrimental consequences on TJ organization and barrier function (Nusrat et al., 1995). SV40 st-mediated PP2A dysfunction also causes defects in TJ assembly by promoting F-actin reorganization in MDCK cells through its action on Rho-GTPases. It reduces expression levels of RhoA, resulting in a loss of stress fibers (Nunbhakdi-Craig et al., 2003). Similarly, PP2A inhibition induces a loss of stress fibers in microcystin-LR treated lung and liver cancer cells; it stimulates the phosphorylation of ezrin, a protein linking the plasma membrane to F-actin, and the actin-regulatory protein, VASP (Wang et al., 2017). Deregulation of PP2A also enhances levels of Rac1 and Cdc42, leading to enhanced membrane ruffling and filopodia in SV40 st-expressing MDCK cells (Nunbhakdi-Craig et al., 2003). Significantly, the binding of active Cdc42 to Par-6 in the PAR complex is essential for establishment of cell polarity; expression of an active mutant of Cdc42 induces TJ disruption by perturbing the normal co-localization of Par-3 and ZO-1 at the TJ (Joberty et al., 2000).

### Microtubule Dynamics Are Intimately Regulated by PP2A

Microtubules interact with and are essential for the biogenesis, organization, integrity, and function of cell–cell junctions. Destabilization of microtubules results in reduced junctional accumulation of ZO-1 and E-cadherin, and altered epithelial barrier function. Conversely, there is increasing evidence that junctional complexes could serve to spatially sequester and control the activity of selected microtubule-associated signaling proteins; this process may be essential to balance cytoskeletal dynamics and junctional integrity (Vasileva and Citi, 2018). Notably, pools of PP2A/Bα holoenzymes are not only associated with TJs, but also directly interact with microtubules in various cell types (Sontag et al., 1995). PP2A is an important regulator of microtubule dynamics. It directly affects the phosphorylation state of microtubules and microtubule-associated proteins that regulate microtubule assembly and stability (Hoffman et al., 2017). In normal (Gurland and Gundersen, 1993) and cancer (Wang et al., 2017) epithelial cell lines, PP2A inhibitory toxins induce the selective depolymerization of stable microtubule populations. This likely contributes to disruption of cell–cell junctions since microtubule stabilization enhances junctional formation (Vasileva and Citi, 2018). Likewise, PP2A/Bα is essential for microtubule stability (Nunbhakdi-Craig et al., 2007). Another PP2A holoenzyme containing the PPP2R5E subunit also binds to and stabilizes microtubule crosslinking factor 1, a cytoskeletal protein that interacts with both F-actin and MTs, thereby affecting epithelial MT organization (Hyodo et al., 2016). Recent findings have also identified a striatin/PP2A/cingulin complex in epithelial cells (Lahav-Ariel et al., 2018). In HEK293T cells, striatin stably associates with the PP2A catalytic subunit and colocalizes with microtubules; its downregulation promotes microtubule destabilization (Kazmierczak-Baranska et al., 2015). Together with the critical role played by cingulin in maintaining the organization of apical microtubules and proper TJ-microtubules interactions (Vasileva and Citi, 2018), it is possible that PP2A/striatin could participate in the regulation of cingulin and microtubule dynamics.

### The PP2A/Intermediate Filament Connection

Desmosomes are linked intracellularly to the IF cytoskeleton, which are abundant cytoplasmic cytoskeletal components of mammalian epithelial tissues. They comprise equal amounts of type I and type II keratin polypeptides that critically determine mechanical stability required to maintain tissue architecture and integrity (Hatzfeld et al., 2017). The organization of IFs protects epithelia against ongoing exposure to various types of physical, chemical, or microbial stressors (Geisler and Leube, 2016). There is also an important cross-talk between IFs and the F-actin/microtubule networks that is imperative for cell polarity (Coch and Leube, 2016). Significantly, PP2A is an important regulator of keratins. Incubation of mouse epidermal keratinocytes or mammary epithelial cells with the PP2A inhibitors, OA and calyculin-A, induces the hyperphosphorylation and solubilization of keratin, resulting in desmosomal disassembly and cell rounding (Kasahara et al., 1993; Favre et al., 1997). PP2A can associate with and dephosphorylate keratin 8 (Tao et al., 2006). Lastly, reduced PP2A expression and activity in human pancreatic carcinoma cells leads to phosphorylation and perinuclear reorganization of keratin 8, which changes cell elasticity and mechanic properties, thereby promoting invasion and metastasis (Park et al., 2016).

Altogether, these findings indicate that marked deregulation of PP2A could indirectly affect the integrity of cell–cell junctional structures by affecting cytoskeletal dynamics.

## Conclusion

As an interactor and modulator of cell junctional structures, PP2A is emerging as a central player in epithelial cell–cell adhesion and polarity. How exactly isoform-specific PP2A-junctional interactions occur and are intimately regulated still remain to be elucidated. PP2A modulates the biogenesis and integrity of junctional and polarity complexes via intricate protein–protein interactions, regulation of cytoskeletal dynamics, and direct dephosphorylation of selected junctional proteins on Ser/Thr residues, which together affect their distribution, and function. Of note, the function of many adhesion proteins is also regulated by tyrosine phosphorylation. As both a substrate and known regulator of selected tyrosine kinases, PP2A has the potential to also indirectly affect the tyrosine phosphorylation state of junctional proteins. Based on the proposed tumor suppressor role of PP2A and its inhibition in many carcinomas, there is growing interest in developing “PP2A activating drugs” for cancer treatment (O’Connor et al., 2018). However, PP2A-dependent dephosphorylation events can induce complex and opposite effects on the assembly and stability of the desmosome, AJ and TJ. For instance, PP2A-mediated dephosphorylation of occludin promotes TJ disruption, a hallmark of EMT. Thus, aberrant PP2A activation could promote metastasis. Further research is paramount to understand how PP2A exactly affects the regulation of cell–cell adhesion, which has profound implications for carcinogenesis and tumor progression.

## Author Contributions

All authors listed have made a substantial, direct and intellectual contribution to the work, and approved it for publication.

## Conflict of Interest Statement

The authors declare that the research was conducted in the absence of any commercial or financial relationships that could be construed as a potential conflict of interest.
